# Origins of Callous-Unemotional Behaviours in Infants

**DOI:** 10.1192/bjo.2022.180

**Published:** 2022-06-20

**Authors:** Gloria Cheung, Francesca Whitehead, Elena Geangu

**Affiliations:** 1 University of York, York, United Kingdom; 2 NHS England, London, United Kingdom

## Abstract

**Aims:**

Callous-unemotional (CU) trait is a characteristic of conduct disorder. As CU-like behaviours emerge from early childhood, this could potentially be predicted early on in life. There is debate in whether general or specifically fear expression processing was impaired in those with CU traits. No studies investigated subliminal emotion processing in those with CU traits. Hence, this study addressed two questions. Firstly, we investigated whether attention to general facial expression or fearful expression is related to future CU behaviours. Secondly, we examined whether subliminal emotion processing can predict CU behaviours alongside supraliminal emotion processing by comparing EEG data to CU behaviours.

**Methods:**

We performed EEG on 7 months old infants using fearful and happy faces as stimuli to investigate whether attention bias to general facial expression or fearful expression is related to future CU behaviours through the Nc component (300–600ms). We also used both subliminal and supraliminal eliciting techniques to determine whether there are any differences in terms of prediction of CU behaviours. The ERP data were then compared with behavioural data, including aggression and empathy scores, collected when the participants reach 14 to 18 months old through the infant-toddler version of the Multidimensional Assessment of Preschool Disruptive Behavior (MAP-DB) and the infant empathy and prosocial behaviour (IEPB) questionnaires.

**Results:**

A total of 18 infant participants were included in our analyses. There is a significant interaction between emotion and empathy for the Nc component, but not aggression. Infants with low empathy paid less attention to fearful facial expressions compared to happy facial expressions while those with high empathy paid more attention to fearful facial expressions compared to happy facial expressions. Moreover, subliminal and supraliminal emotion processing had similar ERP eliciting ability.

**Conclusion:**

Our study showed those with less empathy have a different pattern of attention bias to emotional expression and are less sensitive to fear emotion. Attention bias to emotional expression during infancy could be used to predict CU behaviours during toddlerhood. Being able to predict CU behaviours before their occurrence could help identify those in need of early intervention and help identify potential participants for longitudinal studies that could aid the development of interventions and understanding of CU behaviours. Furthermore, subliminal and supraliminal emotion processing has a similar predicting ability for CU behaviours. This is the first study that investigated subliminal emotion processing in infants with CU behaviours. Future studies would need to include a larger sample size to verify our findings.

